# Exploration and Practice of Maker Education Mode in Innovation and Entrepreneurship Education

**DOI:** 10.3389/fpsyg.2020.01626

**Published:** 2020-09-10

**Authors:** Yan Yang

**Affiliations:** Department of Physics, Luliang University, Luliang, China

**Keywords:** positive psychology, maker education, innovation and entrepreneurship, positive psychological quality, problems and countermeasures

## Abstract

This study was conducted with the purpose of exploring the impact of positive entrepreneurial psychological quality in innovation and entrepreneurship education, as well as the development of maker education in colleges and universities. The questionnaire survey method – *The Positive Mental Characters Scale for Chinese College Students* – and the SPSS 26.0 mathematical statistical analysis software were adopted to analyze and characterize the development of innovation and entrepreneurship education in colleges and universities, as well as the practice of maker education. The results show that there are differences in the factors that affect the positive entrepreneurial psychological quality of college students studying different majors in the liberal arts and sciences. Family economy has the most obvious impact on liberal arts students, sports activities have the most obvious impact on science students, has and grades have the most obvious impact on engineering students; the average score of college students’ innovation and entrepreneurship ability is around 3.0, showing that the overall innovation and creativity ability is general. Furthermore, there are differences in the development of the maker education model between the eastern and western universities. Overall, the maker faculty of eastern universities are more complete, with a larger number of professors, associate seniors, and intermediate teachers. In addition, the investigation on the positive entrepreneurial psychological quality shows a positive effect on cultivating students’ healthy entrepreneurial quality as well as promoting the development and practice of maker education.

## Introduction

Society is in a period of mass innovation and entrepreneurship. In terms of promoting national economic growth, creation, and innovation are key influencing factors. Similarly, they are vital in terms of promoting innovation and entrepreneurship education and improving the innovation and entrepreneurship activities of college students (Kim and Ryu, 2017; Patel et al., 2018). After the international financial crisis, all major economies in the world began to regard innovation as a main strategy to develop advanced technology and improve international competitiveness. During this important period, it is essential to promote coordinated development between universities and society (Bracale et al., 2017; Gillis et al., 2017). At present, higher education is faced with challenges, such as outdated training modes, and the demand for innovative and entrepreneurial talents is increasing day by day. Higher requirements are put forward for the cultivation of innovative and entrepreneurial talents (Faria et al., 2018). Higher education is in urgent need of educational innovation. In this context, the maker education model was developed, a model that emphasizes sharing and practice and focuses on solving practical problems. Integrating the new thinking of maker education with the innovation and entrepreneurship education in colleges and universities can promote the implementation of innovation and entrepreneurship education in colleges and universities and improve the level of innovation and entrepreneurship. Moreover, it also promotes the transformation and innovation of the traditional education mode of colleges and universities, and integrates well with the new mode of social and economic development, thereby achieving a new development situation of mutual benefit, coexistence, and win-win between colleges and society (Bhilwar et al., 2017). At present, many American libraries have participated in maker education activities. A school in Chicago regards maker education as the core concept of school education (Fleisch et al., 2017). Through the establishment of a maker education base alliance, some universities in China have introduced maker spaces, innovation laboratories, and other practice platforms into university education (Mcnamara, 2017).

College students are the main recipients of innovation and entrepreneurship education in colleges and universities. The cultivation of positive psychological qualities in college students is one of the most important factors of ideological and political education in colleges and universities. Accordingly, the improvement of positive psychological qualities of college students is a very important premise whether it promotes the level of mental health of college students or cultivates the sound personality of innovative talents (Bravo et al., 2017; Cornish et al., 2017; Mortier et al., 2017). At the end of the 20th century, Martin Seligman, an American psychologist, put forward the theory of positive psychology to study psychology from a positive perspective, taking the discovery of individual advantages and virtues as an important measure to stimulate people’s enthusiasm and improve their happiness. Meanwhile, the development of positive psychology can play an important role in cultivating the positive psychological quality of college students (Stoner et al., 2017; Otto et al., 2017). From the perspective of innovation and entrepreneurship education, it is crucial to establish and cultivate students’ positive psychological quality.

In summary, there are relatively few research results on the application and practice of the maker education model and few research results on the impact of the psychological quality of college students’ positive entrepreneurial on innovation and entrepreneurship behavior. Based on this, the concept of positive entrepreneurial psychological quality was introduced into the research through a questionnaire survey combined with mathematical statistics that focused on the relationship between the psychological quality of college students’ positive entrepreneurship and the development of maker education. In this way, this study may provide a reference for the application and practice of positive psychology in the transformation and improvement of innovation and entrepreneurship education mode in colleges and universities against the background of the internet.

## Literature Review

### International Research Progress

From the perspective of positive psychology, Sparks et al. (2017) explored and analyzed the role of teachers in shaping students’ physical classroom experiences based on self-determination theory. It was found that the relevant supporting teaching behaviors of physical education teachers can improve students’ classroom experiences. Based on the mixed effect regression model, Tingey et al. (2020) found that entrepreneurial education interventions are greatly significant for promoting the development of youth groups. Laudano et al. (2019) discussed the entrepreneurial intentions, psychological factors, and environmental factors of entrepreneurial university female groups, and found that the entrepreneurial university has a significant influence on female entrepreneurial attitudes and entrepreneurial intentions, and the main differences are related to psychological factors. Weiner et al. (2018) expounded the relevant theories of maker education and developed a new framework for constructing maker spaces, providing a good perspective for educators to implement related projects in maker spaces. Roldan et al. (2018) discussed the impact of the construction and expansion of the university maker space on female engineering college students and provided design principles for the establishment of a university maker space.

### Research Progress in China

By combining the current status of college students’ innovation and entrepreneurship with the development of a new engineering education industry, Zhao et al. (2018) proposed a cognitive system of innovation and entrepreneurship practice with new characteristics. This system is of great significance in improving the innovation and entrepreneurship of new engineering education as well as high-tech application and innovative talents. After exploring the relationship between the internship quality, entrepreneurial intention, and entrepreneurial feasibility of engineering graduates from two research universities in China based on the structural equation model, Yi (2018) concluded that there are significant differences in the family characteristics and entrepreneurial experience of gender groups, which provides new insights in enhancing the entrepreneurial ability of college students. Xu and Li (2018) explored the relationship between college types with innovation and entrepreneurship education, and concluded that there is the greatest gray correlation between the type of colleges and the entrepreneurial competition award, and the gray correlation value of students who choose self-employment is the smallest.

In summary, there have been many research achievements on innovation and entrepreneurship education, the maker education model for colleges, and the innovation and entrepreneurship education based on a psychological level. The relevant research in this field began earlier in places around the world other than China. However, from the perspective of positive psychology, there is relatively little research on the maker education model. As a result, taking positive psychology into consideration, the exploration and practice of maker education in college innovation and entrepreneurship education were discussed.

## Materials and Methods

### Theoretical Basis of Positive Psychology

Positive psychology is a branch of psychological research that became popular in the United States in the 1990s. It focuses on psychology research that ranges from “psychological problems” to “people’s positive power.” It also discusses the conditions and processes conducive to the optimization of individuals, groups, and institutions, and focuses on people’s virtues, subjectivity, and other positive qualities. Martin Seligman, a representative of the field, regards positive psychology as a discipline to study the positive qualities of human development and virtue (Black et al., 2017; Costa et al., 2017). Compared with traditional psychological research, positive psychology has expanded the field of psychological research and overcome the problem of only focusing on the negative aspects of research, including psychological problems and mental diseases. In the research on psychological problems, positive psychology starts from the potential positive factors that people have, and advocates to deal with the problems positively, to explore people’s potential positive power, and actively analyze people’s inner world, thereby promoting the formation of positive psychological qualities (Ciere et al., 2017; Duncan et al., 2017). In terms of research methods, positive psychology, based on absorbing traditional psychological research methods, again combines empirical analysis, phenomenology, and other research methods, including empirical and non-empirical research methods. Regarding research content, positive psychology mainly studies content that includes positive emotions, positive personality traits, and positive social environments, among which the research based on positive psychological environments benefits from regulating positive emotions and cultivating positive psychological qualities, and then promotes the construction of a harmonious social environment. This branch of psychology helps college students to face problems and challenges in the process of innovation and entrepreneurship with a positive attitude. It is essential in realizing successful entrepreneurship, acquiring happiness, improving the life value of college students. It also contributes to the exploration and practice of innovation and entrepreneurship education mode in colleges and universities. Hence, in the process of carrying out innovation and entrepreneurship activities in colleges and universities, it is of great significance to focus on and cultivate the psychological quality of college students’ entrepreneurship.

### Positive Psychological Quality of College Students’ Entrepreneurship

Psychological quality is the representation of a stable psychological feature, an organic whole formed of cognition, emotion, will, and personality, which guides people’s psychology and practical activities (Ranasinghe et al., 2018; Dougherty et al., 2019). Based on this, in the innovation and entrepreneurship activities for college students, entrepreneurial psychological quality is a comprehensive psychological quality formed and developed by individuals under the comprehensive interaction of education and environment. It is a comprehensive and stable way to adjust individual psychological and behavioral characteristics for specific entrepreneurial activities, such as entrepreneurial consciousness, entrepreneurial ability, entrepreneurial will, entrepreneurial personality, and other psychological characteristics (Criaco et al., 2017). The entrepreneurial psychological quality of college students is a stable and individual psychological characteristic which is gradually established during the individual growth process of college students. Specifically, it is formed based on the interaction between environmental impact, education, and students themselves, and it is demonstrated when undertaking entrepreneurial activities. Guided by positive psychology, to fully develop all the leading forces in colleges and universities and actively integrate all the effective forces of society, family, and individuals, are the main measures used to cultivate the psychological quality education of entrepreneurial college students. Positive psychological quality is composed of six dimensions: cognition, interpersonal, emotion, justice, moderation, and transcendence. These in turn are characterized by multiple dimensions and levels. In the process of training college students to develop the psychological quality of positive entrepreneurship, the key is the cultivation of college students’ awareness of active entrepreneurship, good entrepreneurial ability, tenacious entrepreneurial will, and unique entrepreneurial personality. Based on *The Positive Mental Characters Scale for Chinese College Students* (Guo, 2016), Figure 1 shows the dimensions and structure of college students’ positive psychological quality of entrepreneurship.

**FIGURE 1:**
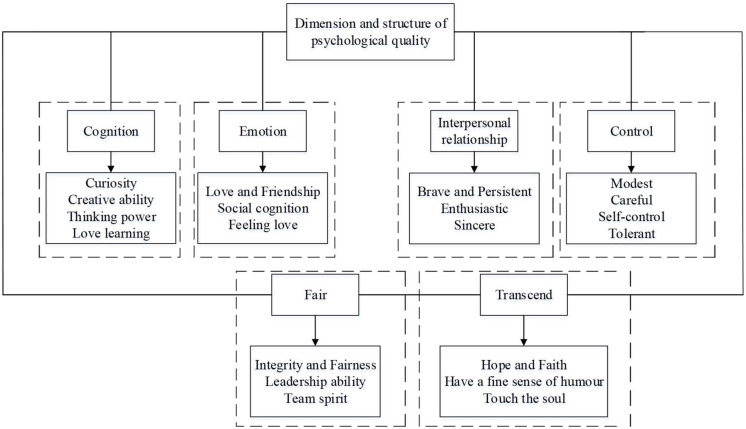
Dimensions and structure of college students’ positive psychological quality of entrepreneurship.

### Internet-Based Maker Education Model

As information technology and the internet develop quickly in today’s society, the deep integration of information technology, the internet, and education is the general trend against which the rise and development of maker education is a typical representative. Maker education is originated from the rise of the word “maker.” Some scholars think that maker refers to people who are eager to transform ideas and creativity into reality due to their own interests, hobbies, and specialties. Creativity is the result of wisdom, which is characterized by individuation and originality. Being able to solve the problems encountered in practice is a response to personal thoughts, rather than a simple act of copy and imitation. The so-called “products” of makers are actually all kinds of creative works (Liu H. et al., 2019). At present, the makers in the development stage mainly include social makers and student makers. Although makers are not necessarily entrepreneurs, there is no denying that the maker movement is complementary to the encouragement of innovation and entrepreneurship policies in China. Compared with entrepreneurship, makers emphasize their interests and hobbies, and regard them as the purpose of practice and creation. Makers’ products can be presented in physical objects or virtual objects, and their subjects can also be people who are good at some kind of software creation or other intangible services. Their works do not take commercial interests as the main purpose. The development of maker education in colleges and universities is mainly studied against the background of the internet, focusing on the maker of college students. Compared with social makers, regarding practical creation, college makers focus on interests and hobbies and have more creative ideas and obvious educational characteristics, and some of them become entrepreneurs after graduation.

“Maker education” is developed on the basis of maker movement and integrates “maker” and “education.” Generally speaking, it can be considered that the purpose of maker education in colleges and universities is to cultivate maker-quality talents (Fleisch et al., 2017). Among them, maker literacy is an expression of ability. People with this ability must have team consciousness and a focus on sharing. Various technical means can be used creatively, thus achieving creation through individual practice or team cooperation and utilizing analysis and problem-solving. The recipients of maker education can be social people or students in school, while the recipients of college maker education can be college students or teachers engaged in maker education. Maker education in colleges and universities has characteristics reflective of the times. It is an educational reform that integrates modern information technology and school education and teaching in the internet environment. Maker education in colleges and universities is an educational tool that cultivates the maker spirit of students, applies information technology, and is in a continuous process of devlopment. It embodies the cooperative inquiry education, which is not limited to a single subject or major but can be completed through the cooperation of interdisciplinary cooperative learning and team cooperation. Maker education in colleges and universities is an educational model formed by combining innovation and entrepreneurship education with higher education. In this development model, students’ innovation ability and entrepreneurship awareness and abilities are expected to achieve synchronous improvement. Therefore, it is particularly important for the cultivation of college students’ positive psychological quality of entrepreneurship. Among them, the differences between the college maker education classroom and the traditional classroom, the elements of college maker education, and the classification of maker space are shown in Table 1.

**TABLE 1:** Comparison and summary of the relevant components of maker education.

Essential factor	Specific categories				
	Classroom Center	Teaching methods	Learning style	Teaching characteristics	Learning objectives
Traditional classroom	Teacher centered	Indoctrination	Hear	Attach importance to books and theories	Knowledge
		Cramming	Recite		
			Remember		
Maker classroom	Student centered	Guided mode	Do	Emphasis on creativity and Practice	Ability and Knowledge
		Open type	Think		
			Say		
Five elements of Maker Education	Maker education course				
	Students in Maker				
	Space in Maker				
	Activities in Maker				
	Maker education teacher				
Space in Maker	Commonweal: Serving makers and ordinary people				
	Educative nature: Serving students				
	Marketability: Serving all people				

### Research Design

(1) Research methods and sample selection

To analyze and study the current situation of college students’ positive psychological quality of entrepreneurship, various types of eastern and western regional colleges and universities were selected, including Beijing University of Aeronautics and Astronautics, Xi’an Jiaotong University, Northwest Normal University, Central South University, Central China Normal University, Dalian Maritime University, and other universities, with the purpose of completing the data collection in the form of a questionnaire. The questionnaire involves entrepreneurial consciousness, entrepreneurial ability, and the entrepreneurial personality of college students. In this survey, the privacy of the respondents was protected, the data was collected anonymously, and the data was promised to be collected only for academic research purposes. This data collection work is mainly in the form of an online electronic questionnaire, aided by “wenjuanxing” (a professional online questionnaire survey platform, which was used to provide users with powerful and humanized online design questionnaire, data collection, custom reports, survey results analysis series of services) to complete the distribution of the questionnaire. The recovered questionnaire, aided by “wenjuanxing,” was used to complete the preliminary statistics and analysis of the data. A Likert scale was used to evaluate the frequency of events related to innovation and entrepreneurship from entrepreneurship awareness, entrepreneurship ability, and entrepreneurship personality, specifically on a scale of “highly disagree,” “not quite disagree,” “uncertain,” “agree more” and “totally agree” (Yen et al., 2018).

(2) Data Analysis Method

Aimed at the research of college students’ positive psychological quality of ntrepreneurship and the development of college maker education, *The Positive Mental Characters Scale for Chinese College Students*, compiled by Meng Wanjin and Guanqun, was used for investigation and analysis. The scale contains 62 questions in total. The reliability test and principal component factor analysis suggest that the scale has good reliability and validity. It is applicable to the large-scale survey of the development of the positive entrepreneurial psychological quality of Chinese college students.

To analyze and process the data, SPSS statistical analysis software was selected, and a mathematical statistical method was applied. This approach is widely used to measure reliability and validity, and multiple linear analysis was used to analyze the data from the questionnaire survey. This process lays a foundation for the follow-up discussion of the development status and existing problems of college students’ innovation and entrepreneurship education. Furthermore, the relationship between the psychological quality of college students’ positive entrepreneurship and the maker education model was examined.

## Results

### Statistics of Questionnaire Survey Results

The demographic characteristics of research samples corresponding to the questionnaire survey are shown in Table 2 below.

**TABLE 2:** Demographic characteristics of research samples.

Demographic variables	Category	Proportion (%)
Gender	Male	44.98
	Female	55.02
School name	Beijing University of Aeronautics and Astronautics	15.49
	Xi’an Jiaotong University	17.30
	Northwest Normal University	10.10
	Central South University	7.11
	Central China Normal University	8.23
	Dalian Maritime University	6.29
	Shanxi University of Finance and Economics	12.45
	Shanxi Normal University	13.26
	Hunan University	5.21
	South China Normal University	4.56
Educational background	Master degree or above	44.77
	Undergraduate	55.23
Major studied	Liberal arts	27.1
	Science	33.5
	Engineering	39.4
Grade	Freshman	6.1
	Sophomore	12.3
	Junior	22.3
	Senior	21.4
	First-year graduated school student	5.9
	Second-year graduated school student	17.21
	Third-year graduated school student	14.8

In this study, 500 college students from diverse majors were selected to carry out the questionnaire survey, and 480 questionnaires were finally recovered. To ensure the validity of the questionnaires used for data analysis, invalid questionnaires among the recovered questionnaires were eliminated. The corresponding effective questionnaire screening standard was that the corresponding topic option did not have too many consistent situations, so it was presented in a more real state. Based on this, the number of valid questionnaires that could be used in this study was 450. The valid recovery rate of the questionnaire was 96%, and the efficiency was 90%. In general, the samples selected cover different genders, majors, and grades, which lays a good foundation for the universal and scientific follow-up study.

### Analysis of the Psychological Quality of College Students’ Positive Entrepreneurship

**FIGURE 2:**
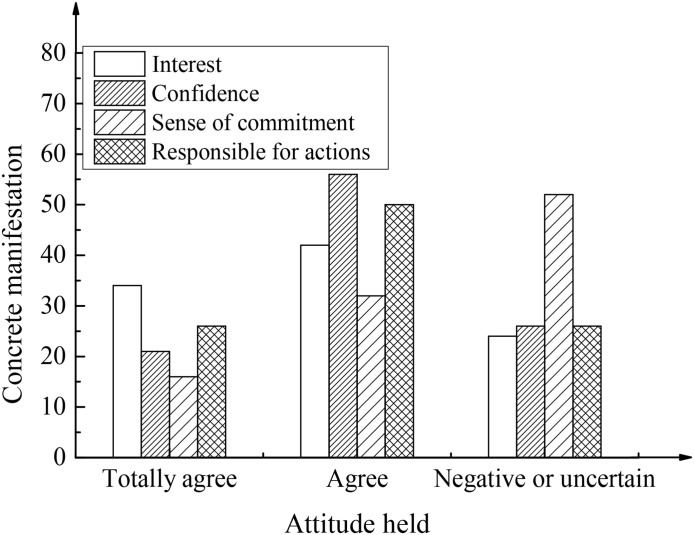
Advantages of positive psychological quality of college students’ entrepreneurship.

To analyze and study the psychological quality of college students’ positive entrepreneurship, the advantages of college students’ positive psychological quality of entrepreneurship were analyzed based on the results of the questionnaire survey. The corresponding analysis results are shown in Figure 2.

Figure 2 shows that in the two sectors of “totally agree” and “agree,” 76.34% of the students are interested in entrepreneurship, 75.44% of the students are confident in solving the difficulties encountered in the process of entrepreneurship, 82.46% of the students are able to face setbacks positively, and 75.11% of the students are able to bear the consequences of their own behaviors bravely. Most college students are interested in and confident in entrepreneurship and can be responsible for their own behavior. Meanwhile, 52.6% of the students choose “totally disagree,” “disagree,” or “uncertain” regarding whether they can undertake individual, family, and social responsibility simultaneously, which shows that college students have insufficient awareness of social responsibility.

**FIGURE 3:**
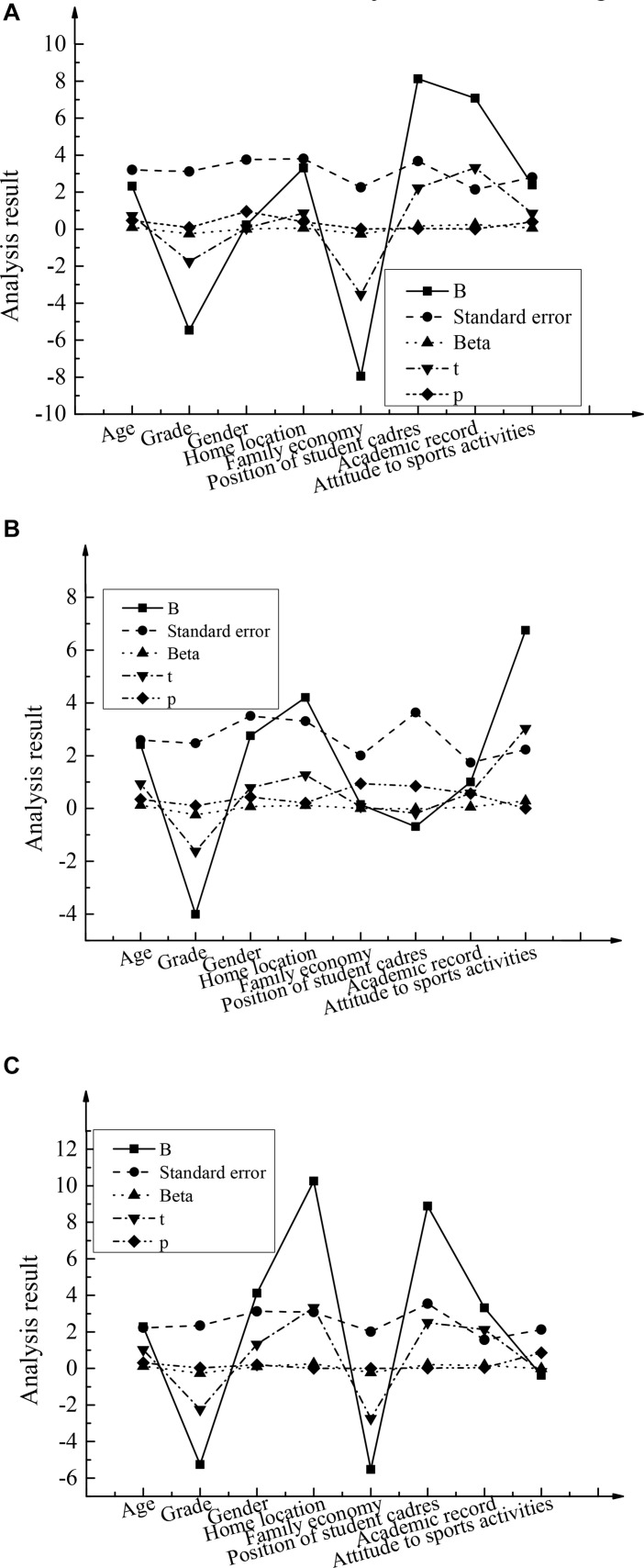
The results of linear regression analysis of demographic characteristics: **(A)** Liberal arts; **(B)** Science; **(C)** Engineering (B: partial regression coefficient, Beta: standardized partial regression coefficient, t: statistics of regression coefficient test, P: significance level).

Then, to get the best data results, the descriptive analysis and statistical analysis were carried out by using mathematical statistics methods. Combined with *The Positive Mental Characters Scale for Chinese College Students*, the linear regression analysis was made for the demographic characteristics of the positive psychological quality of entrepreneurship of college students in the same major, as shown in Figures 3A–C.

Figure 3 shows that college students majoring in liberal arts have a significant influence of 0.01 and 0.05 levels on their positive psychological quality of entrepreneurship in terms of family economy, academic performance, and the employment of student cadres. It is specifically shown as the significant level P of family economy and academic performance for 0.001, and the employment of student cadres for 0.029; the beta coefficients of three corresponding factors are respectively -0.26, 0.25, and 0.16. It can be seen that family economy has the greatest impact on students’ positive psychological quality of entrepreneurship, while other factors have no significant impact. For the college students majoring in science, their attitude toward sports activities has the greatest impact on their positive psychological quality of entrepreneurship; the corresponding beta value is 0.29, while the impact of other factors is not significant. However, for college students majoring in engineering, their grades, family location, family economy, employment of student cadres, and academic achievements have significant influence on their positive psychological quality of entrepreneurial at 0.01 and 0.05 levels, among which grade has the greatest influence with a Beta coefficient value of −0.27, followed by family location (Beta value is 0.26).

### Problems and Countermeasures of Innovation and Entrepreneurship Education

**FIGURE 4:**
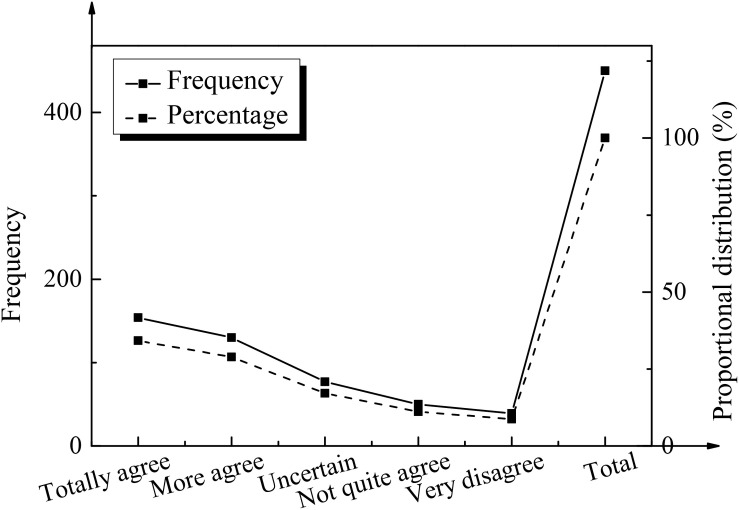
The statistical results of curriculum setting of innovation and entrepreneurship education in colleges and universities.

**FIGURE 5:**
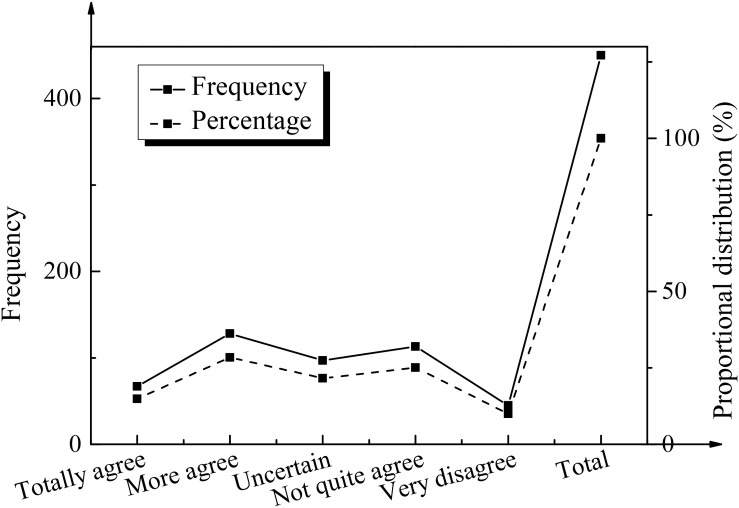
The statistical results of classroom setting and concept penetration of innovation and entrepreneurship in colleges and universities.

To analyze and study the problems and countermeasures in the current innovation and entrepreneurship education in colleges and universities in China, based on the research results of the questionnaire, the analysis of the innovation and entrepreneurship ability of college students is shown in Table 3. The analysis results of the development and importance of innovation and entrepreneurship education in colleges and universities are shown in Figures 4, 5, respectively.

**TABLE 3:** Analysis of the innovation and entrepreneurship ability of college students.

Constituent elements	Average entrepreneurial ability	Basic entrepreneurial ability	Core entrepreneurial ability	Social coping ability
*N*	450	450	450	450
Mean	3.308	3.731	3.568	3.485
Std. Deviation	0.783	0.701	0.889	0.654
Std. Error Mean	0.039	0.036	0.045	0.033

The analysis of the data shows that the average score of innovation and entrepreneurship ability of college students is *M* = 3.32 ± 0.77. It is embodied in the basic innovation and entrepreneurship ability score *M* = 3.72 ± 0.78, the core innovation and entrepreneurship ability score *M* = 3.58 ± 0.88, and the social coping ability score *M* = 3.47 ± 0.66. Generally speaking, compared with the average value of 3, the innovation and entrepreneurship ability of college students is slightly higher. The overall innovation and entrepreneurship ability of college students is general, so it is necessary to strengthen the innovation and entrepreneurship education. By analyzing the data shown in the figure, it can be found that the proportion of “totally agree” and “agree” is 73.2% and the proportion of “highly disagree” is only 1.8%. The implementation of innovation and entrepreneurship courses in colleges and universities in the survey sample is relatively good. After analyzing whether there is penetration of innovation and entrepreneurship concepts in innovation and entrepreneurship courses in colleges and universities, it was found that the proportion of “totally agree” and “agree” is 43.30% in total, while the proportion of “not quite agree” and “highly disagree” is 35.1% in total, and another 25.1% of students are uncertain. The above result indicates that the innovation and entrepreneurship curriculum in colleges and universities does not penetrate the concept of innovation and entrepreneurship.

### Maker Education Under the Positive Psychological Quality of Entrepreneurship

**FIGURE 6:**
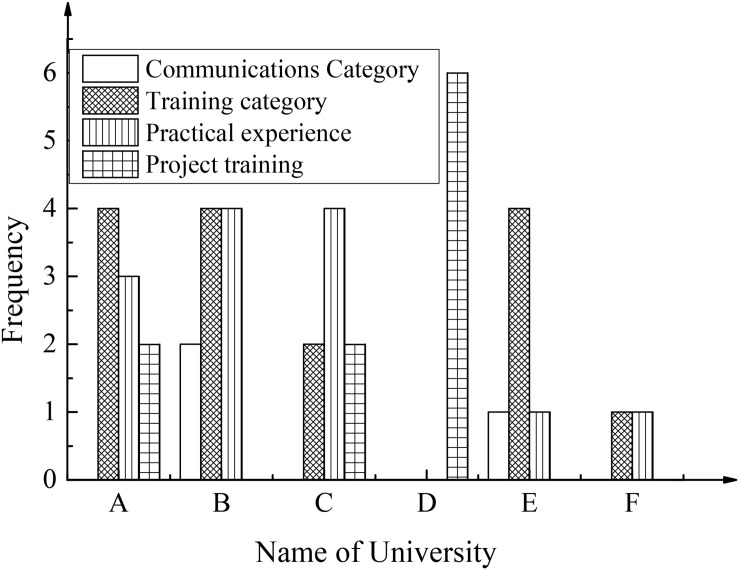
The development of maker activities in colleges and universities (A–F shows the western universities in the sample).

**FIGURE 7:**
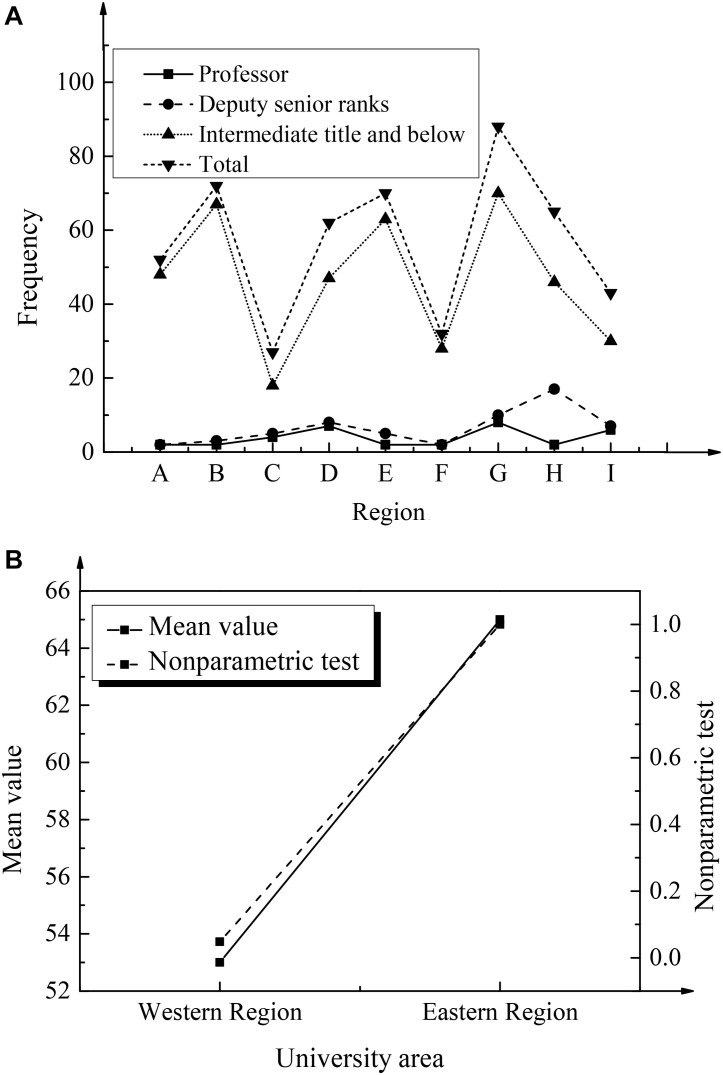
The construction of the faculty of maker education in colleges and universities: **(A)** frequency statistics; **(B)** mean and non-parametric test results (A–F shows the western universities in the sample; G–I represents the eastern universities in the research sample).

To explore the relationship and development between the entrepreneurial psychological quality of college students and maker education in colleges and universities from the perspective of positive psychology, this paper starts from the specific implementation framework of maker education in colleges and universities. Additionally, the research sample of maker activities in colleges and universities and the construction of the maker education faculty were discussed, and corresponding data statistics and analysis was made, as shown in Figures 6, 7, respectively.

From the data analysis, it was found that the sample universities hold relevant maker activities, but they present different types of activities. Among them, training activities can expand communication opportunities between students, experts, scholars, and entrepreneurs. Practical experience activities provide students with opportunities to experience and show themselves, thus promoting students’ creative activities. Exchange activities promote idea exchange among experts, teachers, and students, and inspire students’ innovative thinking. The project training activities can effectively combine professional education and activities, and exercise students’ ability of knowledge application. The above maker activities are of great significance in cultivating students’ positive psychological qualities. The analysis of the construction of the faculty of maker education shows that each university has established a faculty including professors, associate seniors, and middle-level and below teachers. In terms of quantity, the overall average value is 53, among which the average value of the number of associate seniors is eight in western universities and 17 in eastern universities. The average value of the number of teachers with intermediate or lower professional titles is 45 in the western region and 49 in the eastern region. In general, the maker teaching faculty in the eastern universities is more ideal. From the results of the parameter test, there is a significant difference in the number of teachers above associate senior level between the eastern and western universities (*P* = 0.048 < 0.05), and there is no significant difference in the number of teachers with intermediate or lower professional titles between the eastern and western universities (*P* = 1 > 0.05). Generally, in the integration and practical application of the development model of maker education with the innovation and entrepreneurship education in colleges and universities, holding maker activities and constructing teaching staff are important aspects. In the process of promoting the development of maker education model, the measures represented by these two aspects should be emphasized.

## Discussion

In the era of rapid development of the internet and social sciences and technology, information technology can achieve deep integration with education and constantly bring about innovations in education (Feng et al., 2017; Fox, 2017). Different from the traditional education model, maker education conforms to the development of the times, respects the individual differences of students, encourages hands-on practice, and emphasizes the cultivation of students’ innovation ability. As the main carrier of education, it benefits from rich information resources, talent resources, and scientific research achievements of colleges and universities. On this basis, it is necessary to create maker spaces and carry out maker education. It can effectively integrate knowledge and creation, and enhance the atmosphere of innovation and entrepreneurship in colleges and universities, thus promoting the development of innovation and entrepreneurship education in colleges and universities, and providing output power for the continuous cultivation of innovation and entrepreneurship talents. Meanwhile, in the process of innovation and entrepreneurship education, the cultivation of college students’ positive psychological quality of entrepreneurship cannot be ignored. From positive psychology, based on the background of internet development, the concept of college students’ positive psychological quality of entrepreneurship is introduced into the development and improvement of innovation and entrepreneurship education in colleges and universities. In recent years, the success rate of college students choosing to start their own business is relatively low, which is closely linked with the lack of positive psychological quality related to entrepreneurship. It can be seen that, in the process of the development of innovation and entrepreneurship education in colleges and universities, it is necessary to instill and cultivate the positive psychological quality of college students in entrepreneurship.

To change the traditional way of teaching in colleges and universities, and improve the innovation and entrepreneurship level of college students (Bernanke et al., 2017), society should strengthen the propaganda, actively guide, and issue relevant policies to guarantee and promote the school enterprise alliance to solve the problems in current innovation and entrepreneurship education. There is inadequate implementation of policy propaganda, insufficient motivation to participate in innovation and entrepreneurship, unclear understanding of the concept of innovation and entrepreneurship education, an unsound corresponding curriculum system, a lack of teaching staff, insufficient cultural atmosphere for innovation and entrepreneurship, weak family concept, personal awareness, subjective initiative, and a lack of social responsibility awareness (Zheng and Liu, 2020). In addition, colleges and universities should attach importance to students’ innovation and entrepreneurship, clarify the connotation of innovation and entrepreneurship, improve the construction of the curriculum system, improve the level of teaching staff, promote the formation of a strong atmosphere of innovation and entrepreneurship, and focus on the penetration between the development of innovation and entrepreneurship curriculum and concept. What’s more, the individuals should change the traditional employment concept, change the lifelong career concept, establish the modern concept of innovation entrepreneurship, and participate in the practice of innovation and entrepreneurship with positive psychological qualities (Chen, 2019; Wu et al., 2019).

The maker education model based on the maker movement focuses on individuality and emphasizes innovation. In the development and implementation of maker education based on innovation and entrepreneurship education, according to the above data analysis and representation, there are differences between eastern and western universities. Under the influence of the internet development trend, more and more universities are actively exploring and developing maker education (Liu Q. et al., 2019). However, there are still some deficiencies in the development of the Internet + maker education in China, such as the hiring of teachers, the creation of courses, and the establishment of educational platforms. The cultivation of the entrepreneurial psychological quality based on positive psychology is of great significance to the development of innovation and entrepreneurship education in universities against the background of “Internet + maker education” (Shen et al., 2019; Shen and Ho, 2020). The concept of positive psychological quality of entrepreneurship, focusing on the college students, analyzes the problems existing in the innovation and entrepreneurship education based on the questionnaire survey and statistical methods. In addition, it integrates it with the internet and the maker education model in a preliminary manner, aiming to give effective suggestions for the implementation of the maker education model and the development of innovation and entrepreneurship education in colleges and universities (Wu and Song, 2019; Yu et al., 2019).

## Conclusion

In an environment of internet development and innovation and entrepreneurship of the whole society, the exploration and practice of innovation and entrepreneurship education models in colleges and universities was taken as the research object. Additionally, the concept of positive psychological qualities of entrepreneurship was introduced, and the positive psychological qualities of college students’ entrepreneurship was analyzed. Moreover, the relevant factors affecting innovation and entrepreneurship education in colleges and universities were further discussed, and the development of the current maker activities and the construction of the maker teachers were studied. It was found that the factors influencing students’ positive psychological quality of entrepreneurship are different. The effective development of innovation and entrepreneurship education courses is not well integrated with its concepts. The development of maker activities in universities promotes the cultivation of students’ positive psychological qualities of entrepreneurship. Also, there are differences in the construction level of maker teachers between the eastern and western universities. Although the research sample selected in this paper generally covers different types of universities in the east and the west, it is still difficult to guarantee a comprehensive synthesis. Therefore, it is necessary to further deepen and strive to improve the expansion of the data set in future research.

## Data Availability Statement

All datasets generated for this study are included in the article/supplementary material.

## Ethics Statement

The studies involving human participants were reviewed and approved by Luliang University Ethics Committee. The patients/participants provided their written informed consent to participate in this study.

## Author Contributions

The author confirms being the sole contributor of this work and has approved it for publication.

## Conflict of Interest

The author declares that the research was conducted in the absence of any commercial or financial relationships that could be construed as a potential conflict of interest.

## References

[B1] Bernanke J. Galfalvy H. C. Mortali M. G. Hoffman L. A. Moutier C. Nemeroff C. B. (2017). Suicidal ideation and behavior in institutions of higher learning: a latent class analysis. *J. Psychiatr. Res.* 95 253–259. 10.1016/j.jpsychires.2017.09.003 28923719 PMC5826724

[B2] Bhilwar M. Lal P. Sharma N. Sharma N. Bhalla P. Kumar A. (2017). Prevalence of induced abortions and contraceptive use among married women in an urban slum of Delhi. *India. Int. J. Gynaecol. Obstet.* 136 29–32. 10.1002/ijgo.12011 28099705

[B3] Black S. R. Lerner M. D. Shirtcliff E. A. Klein D. N. (2017). Patterns of neuroendocrine coupling in 9-year-old children: effects of sex, body-mass index, and life stress. *Biol. Psychol.* 132:252. 10.1016/j.biopsycho.2017.11.004 29155118 PMC5801078

[B4] Bracale A. Carpinelli G. Falco P. D. (2017). A probabilistic competitive ensemble method for short-term photovoltaic power forecasting. *IEEE Trans Sustain Energy* 8 551–560. 10.1109/tste.2016.2610523

[B5] Bravo A. J. Pearson M. R. Kelley M. L. (2017). Mindfulness and Psychological Health Outcomes: a latent profile analysis among military personnel and college students. *Mindfulness* 9 1–13. 10.1007/s12671-017-0771-5 PMC5800780 29430258

[B6] Chen M. (2019). The impact of expatriates’ cross-cultural adjustment on work stress and job involvement in the high-tech industry. *Front. Psychol.* 10:2228. 10.3389/fpsyg.2019.02228 31649581 PMC6794360

[B7] Ciere Y. Janse M. Almansa J. Visser A. Sanderman R. Sprangers M. A. G. (2017). Distinct trajectories of positive and negative affect after colorectal cancer diagnosis. *Health Psychol.* 36 521–528. 10.1037/hea0000485 28541085

[B8] Cornish P. A. Berry G. Benton S. Barros-Gomes P. Johnson D. Ginsburg R. (2017). Meeting the mental health needs of today’s college student: reinventing services through stepped care 2.0. *Psychol. Serv.* 14 428–442. 10.1037/ser0000158 29120201

[B9] Costa S. D. Théro H. Chambon V. Haggard P. (2017). Try and try again: post-error boost of an implicit measure of agency. *Q. J. Exp. Psychol.* 71 1–28. 10.1080/17470218.2017.1350871 28697690

[B10] Criaco G. Sieger P. Wennberg K. Chirico F. Minola T. (2017). Parents’ Performance in Entrepreneurship as a “Double-Edged Sword” for the Intergenerational Transmission of Entrepreneurship. *Small Bus. Econ.* 49 1–24.

[B11] Dougherty C. M. Burr R. L. Kudenchuk P. J. Glenny R. W. (2019). Aerobic exercise effects on quality of life and psychological distress after an implantable cardioverter defibrillator. *J. Cardiopulm. Rehabil. Prev.* 40 94–101. 10.1097/HCR.0000000000000444 31397768 PMC7004855

[B12] Duncan G. J. Magnuson K. Votruba-Drzal E. (2017). Moving beyond correlations in assessing the consequences of poverty. *Annu. Rev. Psychol.* 68:413. 10.1146/annurev-psych-010416-044224 27648987 PMC6108837

[B13] Faria J. R. Wanke P. F. Ferreira J. J. Mixon F. G. (2018). Research and innovation in higher education: empirical evidence from research and patenting in Brazil. *Scientometrics* 116 487–504. 10.1007/s11192-018-2744-4

[B14] Feng T. Shen X. Huang R. Chen G. H. (2017). Influence of the interannual variation of cross-equatorial flow on tropical cyclogenesis over the western North Pacific. *J. Trop. Meteorol.* 23 68–80.

[B15] Fleisch B. Taylor S. Schöer V. Thabo M. (2017). The value of large-scale randomised control trials in system-wide improvement: the case of the Reading Catch-Up Programme. *S. Afr. J. Educ.* 37 1–13. 10.15700/saje.v37n1a1248

[B16] Fox S. (2017). Mass imagineering: combining human imagination and automated engineering from early education to digital afterlife. *Technol. Soc.* 51 163–171. 10.1016/j.techsoc.2017.09.001

[B17] Gillis D. Nelson J. Driscoll B. Hodgins K. Fraser E. Jacobs S. (2017). Interdisciplinary and transdisciplinary research and education in canada: a review and suggested framework. *Collect. Essays Learn. Teach.* 10:203 10.22329/celt.v10i0.4745

[B18] Guo L. (2016). Research of confucianism education method in chinese college students’ ideological and political education. *Creat. Educ.* 7 1051–1055. 10.4236/ce.2016.77109

[B19] Kim Y. Ryu M. H. (2017). Towards entrepreneurial organization: from the case of organizational process innovation in Naver. *Proc. Comput. Sci.* 122 663–670. 10.1016/j.procs.2017.11.421

[B20] Laudano M. C. Zollo L. Ciappei C. Zampi V. (2019). Entrepreneurial universities and women entrepreneurship: a cross-cultural study. *Manag. Decis.* 57 2541–2554. 10.1108/md-04-2018-0391

[B21] Liu H. Zhu W. Dong H. Ke Y. (2019). An adaptive ball-head positioning visual servoing method for aircraft digital assembly. *Assem. Autom.* 39 287–296. 10.1108/aa-05-2018-066

[B22] Liu Q. Cheng Z. Chen M. (2019). Effects of environmental education on environmental ethics and literacy based on virtual reality technology. *Electron. Libr.* 37 860–877. 10.1108/EL-12-2018-0250

[B23] Mcnamara L. (2017). Water quality modelling for decision-making: the drinking-water watersheds of Sydney. Australia. *J. Financial Serv. Res.* 65 236–243.

[B24] Mortier P. Cuijpers P. Kiekens G. Auerbach R. P. Demyttenaere K. Green J. G. (2017). The prevalence of suicidal thoughts and behaviours among college students: a meta-analysis. *Psychol. Med.* 48 1–12. 28805169 10.1017/S0033291717002215

[B25] Otto L. R. Sin N. L. Almeida D. M. Sloan R. P. (2017). Trait emotion regulation trategies and diurnal cortisol profiles in healthy adults. *Health Psychol.* 37:301. 10.1037/hea0000564 29172603 PMC5837936

[B26] Patel P. S. Chung K. Y. Kasrai L. (2018). Innovate global plastic and reconstructive surgery: cleft lip and palate charity database. *J. Craniofac. Surg.* 29:1. 10.1097/SCS.0000000000004374 29485559

[B27] Ranasinghe N. Devanarayana N. M. Rajindrajith S. Perera M. S. Nishanthinie S. Warnakulasuriya T. (2018). Functional gastrointestinal diseases and psychological maladjustment, personality traits and quality of life. *BMC Gastroenterol.* 18:33. 10.1186/s12876-018-0760-8 PMC5830068 29486708

[B28] Roldan W. Hui J. Gerber E. M. (2018). University makerspaceS: opportunities to support equitable participation for women in engineering. *Int. J. Eng. Educ.* 34 751–768.

[B29] Shen C.-W. Ho J.-T. (2020). Technology-enhanced learning in higher education: a bibliometric analysis with latent semantic approach. *Comput. Hum. Behav.* 104:106177 10.1016/j.chb.2019.106177

[B30] Shen C.-W. Min C. Wang C.-C. (2019). Analyzing the trend of O2O commerce by bilingual text mining on social media. *Comput. Hum. Behav.* 101 474–483. 10.1016/j.chb.2018.09.031

[B31] Sparks C. Lonsdale C. Dimmock J. Jackson B. (2017). An intervention to improve teachers’ interpersonally involving instructional practices in high school physical education: implications for student relatedness support and in-class experiences. *J. Sport Exerc. Psychol.* 39 120–133. 10.1123/jsep.2016-0198 28787252

[B32] Stoner C. R. Orrell M. Long M. Csipke E. Spector A. (2017). The development and preliminary psychometric properties of two positive psychology outcome measures for people with dementia: the PPOM and the EID-Q. *BMC Geriatr.* 17:1–11. 28327088 10.1186/s12877-017-0468-6 PMC5361794

[B33] Tingey L. Larzelere F. Goklish N. Rosenstock S. Mayo-Wilson L. J. Pablo E. (2020). Entrepreneurial, Economic, and Social Well-Being Outcomes from an RCT of a Youth Entrepreneurship Education Intervention among Native American Adolescents. *Int. J. Environ. Res. Public Health* 17:2383. 10.3390/ijerph17072383 32244495 PMC7177681

[B34] Weiner S. Lande M. Jordan S. (2018). The engineer of 2020, in the making: understanding how young adults develop maker identities and the implications for education reform. *Int. J. Eng. Educ.* 34 833–842.

[B35] Wu Y. Song D. (2019). “Gratifications for Social Media Use in Entrepreneurship Courses: learners’. Perspective”. *Front. Psychol.* 10:1270. 10.3389/fpsyg.2019.01270 PMC6555126 31214081

[B36] Wu Y. Wu T. Li Y. (2019). Impact of using classroom response systems on students’ entrepreneurship learning experience. *Comput. Hum. Behav.* 92 634–645. 10.1016/j.chb.2017.08.013

[B37] Xu J. Li Y. (2018). Grey incidence analysis model of classification variables and its application on innovation & entrepreneurship education in Jiangsu. *J. Grey Syst.* 30 123–128.

[B38] Yen J. Y. Liu T. L. Chen J. I Chen S. Y. Ko C. H. (2018). Premenstrual appetite and emotional responses to foods among women with premenstrual dysphoric disorder. *Appetite* 125 18–23. 10.1016/j.appet.2018.01.029 29407746

[B39] Yi G. (2018). Impact of internship quality on entrepreneurial intentions among graduating engineering students of research universities in China. *Int. Entrepreneursh. Manag. J.* 14 1071–1087. 10.1007/s11365-017-0491-2

[B40] Yu C. Zhang Z. Lin C. Wu Y. (2019). Can data-driven precision marketing promote user AD clicks? Evidence from advertising in WeChat moments. *Ind. Mark. Manag.* 88, 414–425. 10.1016/j.indmarman.2019.05.001

[B41] Zhao S. Zhang H. Wang J. (2018). Cognition and system construction of civil engineering innovation and entrepreneurship system in emerging engineering education. *Cogn. Syst. Res.* 52 1020–1028. 10.1016/j.cogsys.2018.10.020

[B42] Zheng Y. Liu S. (2020). Bibliometric analysis for talent identification by the subject–author–citation three-dimensional evaluation model in the discipline of physical education. *Libr. Hi Tech* 10.1108/LHT-12-2019-0248 [Epub ahead of print].

